# The antipsychotic drug pimozide promotes apoptosis through the RAF/ERK pathway and enhances autophagy in breast cancer cells

**DOI:** 10.1080/15384047.2024.2302413

**Published:** 2024-02-14

**Authors:** Ge Jiang, Xingzhi Zhou, Ye Hu, Xiaoyu Tan, Dan Wang, Lina Yang, Qinggao Zhang, Shuangping Liu

**Affiliations:** a Chronic Disease Research Center, Medical College, Dalian University, Dalian, Liaoning, China; b Department of Biology, Life Science and Technology College, Dalian University, Dalian, Liaoning, China; c Department of Clinical Laboratory, Xin Hua Hospital Affiliated to Dalian University, Dalian, China

**Keywords:** Breast cancer, Pimozide, RAF/ERK, Apoptosis, Autophagy

## Abstract

The antipsychotic drug pimozide has been demonstrated to inhibit cancer. However, the precise anti-cancer mechanism of pimozide remains unclear. The purpose of this study was to investigate the effects of pimozide on human MCF-7 and MDA-MB-231 breast cancer cell lines, and the potential involvement in the RAF/ERK signaling. The effects of pimozide on cells were examined by 4,5-dimethylthiazol-2-yl-3,5-diphenylformazan, wound healing, colony formation, transwell assays, and caspase activity assay. Flow cytometry and acridine orange and ethidium bromide staining were performed to assess changes in cells. Transmission electron microscopy and monodansylcadaverine staining were used to observe autophagosomes. The cyclic adenosine monophosphate was evaluated using the FRET system. Immunohistochemistry, immunofluorescence, RNA interference, and western blot investigated the expression of proteins. Mechanistically, we focus on the RAF1/ERK signaling. We detected pimozide was docked to RAF1 by Schrodinger software. Pimozide down-regulated the phosphorylation of RAF1, ERK 1/2, Bcl-2, and Bcl-xl, up-regulated Bax, and cleaved caspase-9 to induce apoptosis. Pimozide might promote autophagy by up-regulating cAMP. The enhancement of autophagy increased the conversion of LC3-I to LC3-II and down-regulated p62 expression. But mTOR signaling was not involved in promoting autophagy. The knockdown of RAF1 expression induced autophagy and apoptosis in breast cancer cells, consistent with the results of pimozide or sorafenib alone. Blocked autophagy by chloroquine resulted in the impairment of pimozide-induced apoptosis. These data showed that pimozide inhibits breast cancer by regulating the RAF/ERK signaling pathway and might activate cAMP-induced autophagy to promote apoptosis and it may be a potential drug for breast cancer treatment.

## Introduction

Breast cancer, a frequently diagnosed cancer in females, has long been one of the leading causes of cancer-related death in women. Breast cancer alone accounts for 25% of all cancer cases and 15% of all cancer deaths among females.^1^ Approximately 2.1 million females were estimated to be diagnosed with breast cancer worldwide in 2018, accounting for about one-fourth of cancers in females.^2^ More than 30% of patients with metastatic breast cancer do not respond to first-line chemotherapy based on anthracyclines and taxanes, and their cancers progress in less than a year.^3^ Since the first report of breast cancer treatment with tamoxifen in 1985, a variety of cancer drugs have been developed for breast cancer treatment.^4^ In recent years, the repurposing of approved drugs for the treatment of other diseases has shown efficacy as a new therapeutic approach for new drug development in diseases including cancer.^5^ Several FDA-approved clinical medications have been examined for the treatment of cancer, providing a diverse selection of cancer treatments and better anticancer effects.^6^ For example, the FDA-approved drugs metformin, aspirin, and sulfonamide have exhibited anti-cancer effects.^7–9^ The reuse of existing drugs is more advantageous for safety reasons. Therefore, the continued identification of previously approved drugs that show potent anti-cancer effects will enable new strategies for cancer treatment.

Pimozide, as a D2-type dopamine receptor inhibitor, is a typical antipsychotic drug that is commonly used in the treatment of mental disorders. Recent studies have shown that pimozide also inhibits a variety of cancer cells, such as colorectal cancer, human osteosarcoma, and hepatocellular carcinoma.^10–12^ Pimozide inhibits ERK signaling in human osteosarcoma and can selectively promote autophagy in glioblastoma.^11,13^ Previous studies demonstrated that RAF1 had clinical value and prognostic significance in non-small cell lung cancer patients, and we found that pimozide down-regulated the phosphorylation of RAF1.^14^ Bertolesi et al. reported that pimozide inhibits the growth of MCF-7 breast cancer cells via T-type Ca^2+^ channels and suggested that its growth inhibitory effects may be mediated by additional mechanisms.^15^ However, the precise mechanism of action of pimozide in breast cancer remains unclear.

Previous studies have demonstrated that RAF1 has anti-apoptotic functions. RAF1 is an important protein that interfaces between the RAS and MEK/ERK signaling pathways. RAF1 also exerts its cellular functions through pathways such as the Hippo pathway, which is antagonized by RAF1.^16,17^ RAF1 interaction with MST2 is a link between the MAPK and Hippo pathways.^18–21^ RAF1 activates extracellular regulated protein kinase (ERK), a member of the MAPK family, which is involved in the proliferation, invasion, and migration of breast cancer cells.^22,23^ ERK inhibits autophagy by activating mTOR signaling and down-regulates the anti-apoptotic protein Bcl-xl to promote cell survival.^24,25^ Autophagy plays an important role in maintaining the balance of energy metabolism and cell survival within cells. However, once autophagy is overactivated, apoptosis occurs.^26,27^ Many studies have focused on simultaneously inducing tumor cell apoptosis while interfering with autophagy for the treatment of cancer. As shown in previous studies, Cucurbitacin I promoted apoptosis and pro-death autophagy via ERK/mTOR/STAT3 signaling, and 3β,11-dihydroxy-9,11-secogorgost-5-en-9-one induced apoptosis due to its ability to promote autophagy.^28,29^

This study aimed to investigate the mechanism of pimozide inhibition on human MCF-7 (an estrogen receptor-positive cell line) and MDA-MB-231 (a triple negative cell line) breast cancer cells. For this purpose, the growth progression of the treated cells was assessed. Subsequently, the apoptosis and autophagy-related protein expression were evaluated after being treated. A special focus was placed on the RAF/ERK signaling. To investigate the contribution of RAF1 in autophagy and apoptosis, the introduction of inhibitors was considered an effective strategy. In brief, our work was to provide a rationale for pimozide as one of the potential anti-cancer drug candidates.

## Results

### High RAF1 expression predicts poor prognosis in breast cancer patients

**Figure 1.**
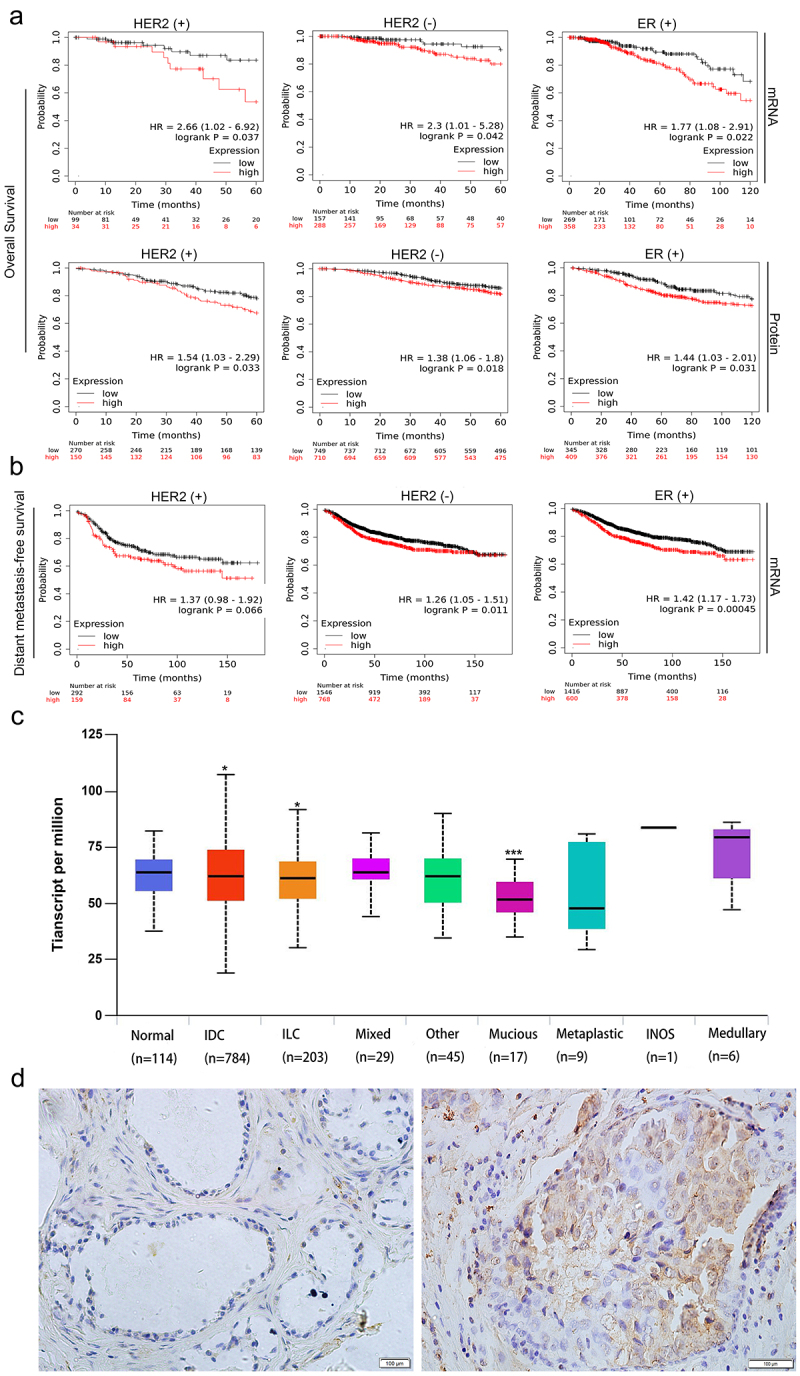
RAF1 is expressed in a variety of breast cancer histological subtypes, and high expression of RAF1 is associated with the prognosis of HER-2 positive breast cancer. (a) the Kaplan-Meier database was used to analyze the relationship between the protein expression or mRNA level of RAF1 and overall survival and **(b)** distant metastasis-free survival in breast cancer patients with HER2 (±) or ER (+) breast cancer. **(c)** analysis of the expression of RAF1 in different histological subtypes of breast cancer based on the ualcan database (http://ualcan.Path.uab.edu/index.Html) (*, *p* < .05, ***, *p* < .001). **(d)** the expression of RAF1 in breast cancer (right) and adjacent tissues (left).

We examined the relationship between RAF1 expression and prognosis in breast cancer patients. As shown in Figure 1A, we analyzed RAF1 mRNA expression levels and protein expression in ER (±), PR (±), and HER2 (±) breast cancer samples using the Kaplan-Meier database.^30^ We found that HER2 (±) breast cancer cases with RAF1 high expression had a shorter survival time (*p* = .037, *p* = .042), and high RAF1 mRNA expression had a shorter survival time (*p* = .033, *p* = .018). ER2 (+) breast cancer cases with RAF1 protein and mRNA high expression had a shorter survival time (*p* = .022, *p* = .031). ER2 (-) and PR (±) breast cancer cases with RAF1 protein and mRNA high or low expression were not related to survival (**Figure S1**). We next examined the association of RAF1 mRNA expression with distant metastasis-free survival in HER2 (±) breast cancer samples. Cases with high RAF1 mRNA expression had a lower distant metastasis-free survival in HER2 (-) breast cancers (*p* = .011), while HER2(+) breast cancer was not related to the distal metastasis-free survival (*p* = .066) (Figure 1B). We also evaluated breast cancers according to histological subtype.^31^ As shown in Figure 1C, RAF1 expression was associated with infiltrating ductal carcinoma, infiltrating lobular carcinoma, and mucinous carcinoma and was not associated with other histological subtypes. We examined the expression of RAF1 in breast cancer and adjacent tissues by immunohistochemistry and found that RAF1 expression was related to HER2 (Figure 1D, Tables 1 and 2). These results suggested that RAF1 expression was associated with a poor prognosis of breast cancer.

**Table 1.** RAF1 protein expression in breast cancer samples and adjacent tissue samples.

Diagnosis	No. of cases	RAF1 expression				Positive rate (%)	Strongly positive rate (%)
		-	+	++	+++		
Breast cancer	104	9	21	56	18	91.3%	71.2%
Adjacent tissues	46	5	13	24	4	89.1%	60.9%

Positive rate: percentage of positive cases with ‘+’, ‘++’, and ‘+++’ staining score; Strongly positive rate: percentage of positive cases with ‘++’ and ‘+++’ staining score.

**Table 2.** Relationship between RAF1 overexpression and the clinicopathological features in breast cancer.

Characteristic	No. of cases	Strongly positive cases (%)	*P* value
Age (years old)			0.0530
≤55	66	38 (57.6%)	
>55	84	35 (41.7%)	
TNM Clinical Stage			.3351
I-II	114	58 (5.9%)	
III-IV	36	15 (41.7%)	
HER2			.0297*
+	45	28 (62.2%)	
-	105	45 (42.9%)	
Tissue			.2302
breast cancer	104	54 (51.9%)	
Adjacent tissues	46	19 (41.3%)	

*p < .05.

## Pimozide inhibits the proliferation and colony formation of breast cancer cells

**Figure 2.**
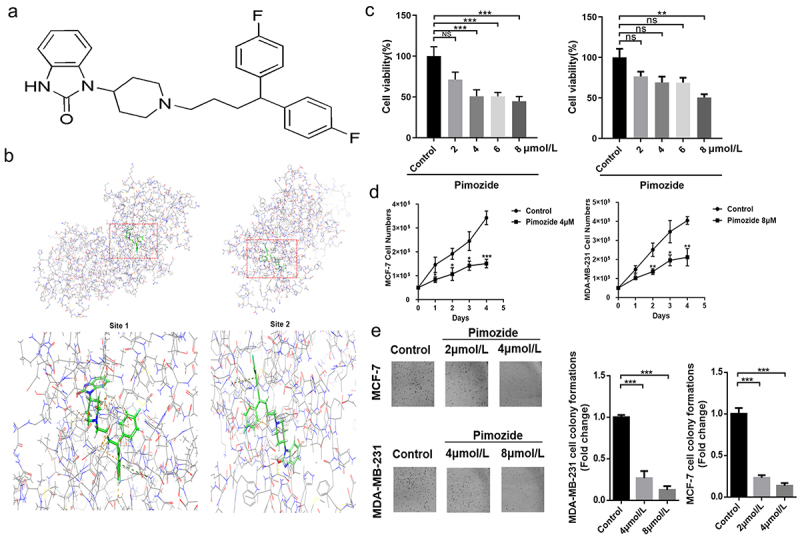
**Pimozide inhibits breast cancer cell proliferation. (a)** the pimozide structure was drawn using ChemDraw. **(b)** pimozide docked with RAF1 and found two binding sites by Schrodinger software, identified as site 1 and site 2. **(c)** MTT assay to assess the effects of different concentrations of pimozide on breast cancer cells (6 × 10^3^ cells/well). **(d)** effect of 4 μM or 8 μM pimozide on the growth of breast cancer cells (5 × 10^4^ cells) within 4 days. **(e)** after treatment with pimozide, the number of clones of breast cancer cells (1 × 10^3^ cells) was reduced. Percent adhesion was normalized to DMSO control for 3 replicate experiments with 3 or more replicate wells (*, *p* < .05, ***, *p* < .001).

Pimozide is a diphenylbutylpiperidine antipsychotic (Figure 2A) that was shown to inhibit the growth of breast cancer MCF-7 cells and our previous study found that pimozide competes with L-lactate for binding to oxygen-regulated protein N-myc downstream-regulated gene 3, an upstream signal of RAF1.^32,33^ Based on these reports, and the discovery in the UALCAN and Kaplan-Meier database, we simulated the binding of pimozide to RAF1 using Schrodinger software. As predicted by Schrodinger software, pimozide might have two binding sites with RAF1: the Site 1 and Site 2 scores were 4.344 and 5.933, respectively (Figure 2B).

We indicated the effect of pimozide on the viability of human breast cancer cells. MCF-7 and MDA-MB-231 cells were treated with pimozide for 24 h and MTT assays were performed. As shown in Figure 2C, pimozide decreased the viability of MCF-7 and MDA-MB-231 cells with IC_50_ values of 4 μM and 8 μM, respectively. Furthermore, the proliferative of breast cancer cells treated with pimozide was down-regulated compared with untreated cells (Figure 2 2D). Our results were similar to previous reports.^34,35^ To confirm the efficacy of pimozide in inhibiting breast cancer cell proliferation, we also performed colony formation analysis. Pimozide treatment resulted in a reduction of the colony formation ability of both MCF-7 and MDA-MB-231 cells compared with the control groups (Figure 2E). Together these data indicated that pimozide inhibited the proliferation of breast cancer cells.

## Pimozide induces apoptosis in breast cancer cells

**Figure 3.**
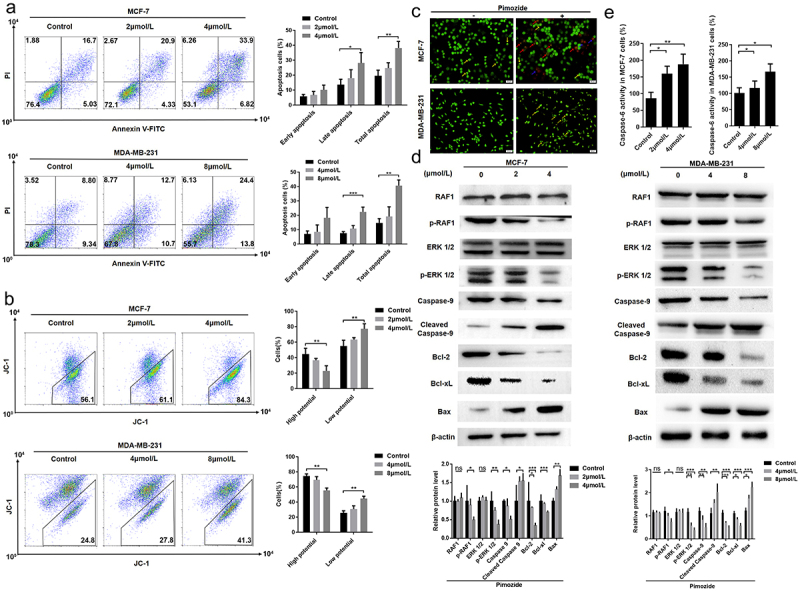
**Pimozide inhibits breast cancer growth through the RAF/ERK signaling pathway**. flow cytometry was used to detect apoptosis **(a)** and mitochondrial membrane potential **(b)** in breast cancer cells at different concentrations of pimozide. **(c)** AO-EB staining to identify normal cells (green), early apoptosis (yellow arrow), late apoptosis (red arrow), dead cells (blue arrow), MCF-7 (1 × 10^3^ cells), and MDA-MB-231 cells (1 × 10^3^ cells) were treated with 4 μM and 8 μM pimozide, respectively. **(d)** Western blot analysis of protein expression increased with the concentration of pimozide. **(e)** the effect of pimozide on the activities of caspase-6 after treating MCF-7 and MDA-MB-231 cells. Summary data from three replicate experiments with three replicate samples, ns means no statistical significance (*, *p* < .05, **, *p* < .01, ***, *p* < .001).

We next examined the mechanism of the anti-proliferative activity of pimozide by performing flow cytometry. We found that pimozide treatment induced a significant increase in the apoptotic cell population in a concentration-dependent manner both in MCF-7 and MDA-MB-231 cells (Figure 3A). A similar result was obtained with the JC-1 analysis. As shown in Figure 3B, as the concentration of pimozide increased, we observed increased numbers of MCF-7 and MDA-MB-231 cells with decreased membrane potential. We further distinguished live cells and cells in different apoptotic stages by AO-EB staining. As shown in Figure 3C, after treatment with 4 μM pimozide, viable apoptotic cells increased in MCF-7 cells, while MDA-MB-231 cells showed an increase in cells in advanced apoptosis after 8 μM pimozide treatment. These data indicated that pimozide-induced apoptosis contributed to determining the antiproliferative effect observed in breast cancer cells.

## The RAF/ERK pathway is involved in pimozide-induced apoptosis

We further explored the mechanism by which pimozide regulates the relationship between RAF1 and mitochondrial membrane potential. Under the stimulation of apoptotic signals, the membrane potential of mitochondria is lost, and the mitochondrial permeability changes. We next examined the expression levels of proteins in the RAF1 pathway and several regulators of mitochondrial permeability by western blot analysis. As shown in Figure 3 3D, while RAF1 and ERK 1/2 expression were unaffected by pimozide, both p-RAF1 and p-ERK 1/2 levels were down-regulated by pimozide in a dose-dependent manner in MCF-7 cells. Consistent with our apoptosis results, the levels of the mitochondrial permeability-related proteins Bcl-2 and Bcl-xl were decreased and Bax was increased in pimozide-treated MCF-7 cells. In addition, the apoptosis initiation protein caspase-9 was down-regulated, and cleaved caspase-9 expression increased with increasing pimozide concentration. And the activities of caspase 6 were enhanced in MCF-7 and MDA-MB-231 cells (Figure 3E). These data confirmed that pimozide inhibited the phosphorylation of RAF1 and ERK 1/2 and induced apoptosis in breast cancer cells.

Previous studies reported that RAF1 regulates the Hippo pathway through its interaction with MST2 to alter cell proliferation, migration, and invasion.^21,36^ We examined the expression of Hippo pathway proteins YAP/TAZ, MST1, and MST2 in MCF-7 cells treated with increasing concentrations of pimozide (**Figure S2**). However, pimozide had no impact on the expressions of these proteins.

## Pimozide promotes autophagy in breast cancer cells

**Figure 4.**
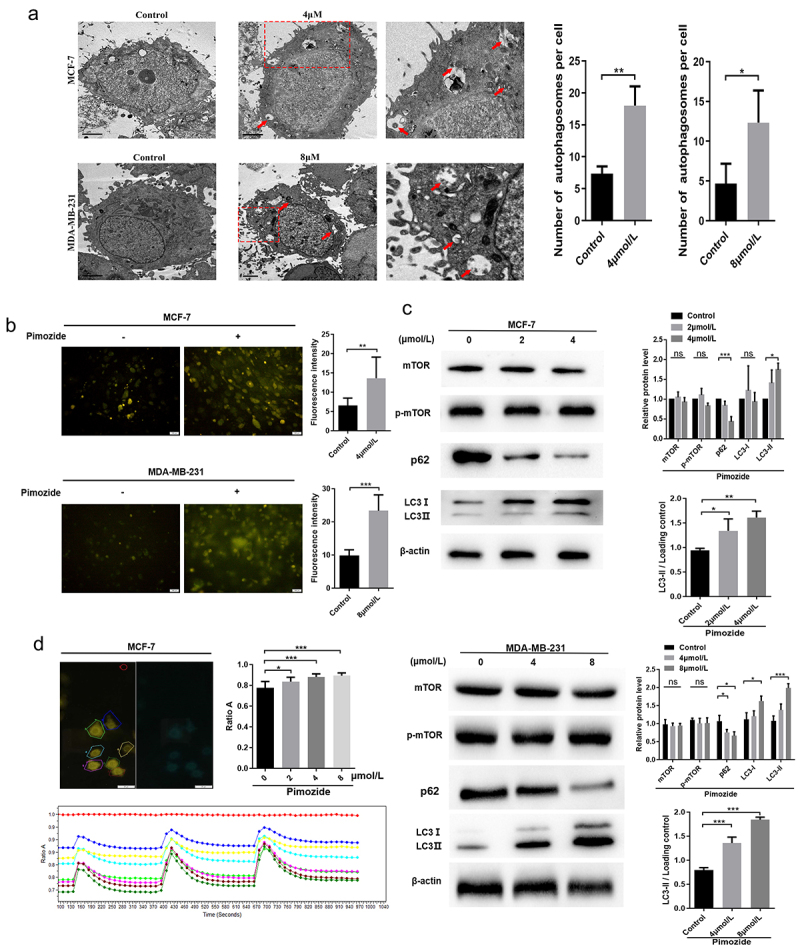
**Pimozide might promote autophagy in breast cancer cells by up-regulating cAMP**. TEM **(a)** and MDC staining **(b)** observed the number of autophagosomes in breast cancer cells after pimozide (4 μM or 8 μM) (red arrows indicate autophagosomes). **(c)** Western blot was used to detect autophagy-related proteins, and β-actin was used as an internal reference. **(d)** detection of changes in cAMP with increasing pimozide concentration based on the FRET system. The left image was the YFP fluorescence signal, the red circle represented the baseline, the other coils surrounded the cell, the right side was the CFP fluorescence signal, and ratio a meant the fluorescence intensity of the CFP was greater than the fluorescence intensity of the YFP. All experiments were performed three times in parallel, ns means no statistical significance (*, *p* < .05, ***, *p* < .001).

Autophagy plays an important role in providing energy for tumor cells. However, hyperactivation of autophagy is often accompanied by the occurrence of apoptosis. Zielke et al. demonstrated that pimozide promotes autophagic-dependent death in glioma cells.^37^ To evaluate the effect of pimozide on the induction of autophagy-dependent cell death in breast cancer cells, MDC staining assay and TEM were used to quantify cells undergoing autophagy. We observed autophagosomes by TEM in breast cancer cells and found that pimozide-stimulated cells showed increased autophagosomes compared with controls (Figure 4A). We further examined changes in autophagosomes by MDC staining analysis and the results were consistent with the TEM results. Treatment of MCF-7 and MDA-MB-231 cells with pimozide for 24 h resulted in enhanced autophagy (Figure 4B). Together these findings indicated that pimozide promoted autophagy in breast cancer cells.

To further research the mechanism involved in pimozide-mediated cell autophagy, we examined the expressions of autophagy-related proteins by western blot. As shown in Figure 4C, the conversion of LC3-I to LC3-II was increased and the level of p62 was significantly decreased in MCF-7 cells treated with pimozide. However, mTOR, an autophagy inhibitor, was not impacted by pimozide treatment (Figure 4C). Previous studies showed that RAF1 interacts with cAMP and upregulation of cAMP can promote autophagy in cells.^38,39^ We used FRET to detect cAMP changes before and after pimozide treatment in live breast cancer cells (Figure 4 4D). The data showed that pimozide promoted the up-regulation of cAMP in a dose-dependent manner, cAMP levels reduced after time but did not return to normal levels within the examined time frame. These data suggested that pimozide might promote autophagy by up-regulating cAMP, but mTOR was not involved in pimozide-mediated induction of autophagy in breast cancer cells.

## RAF1 plays an important role in the anticancer effects of pimozide

**Figure 5.**
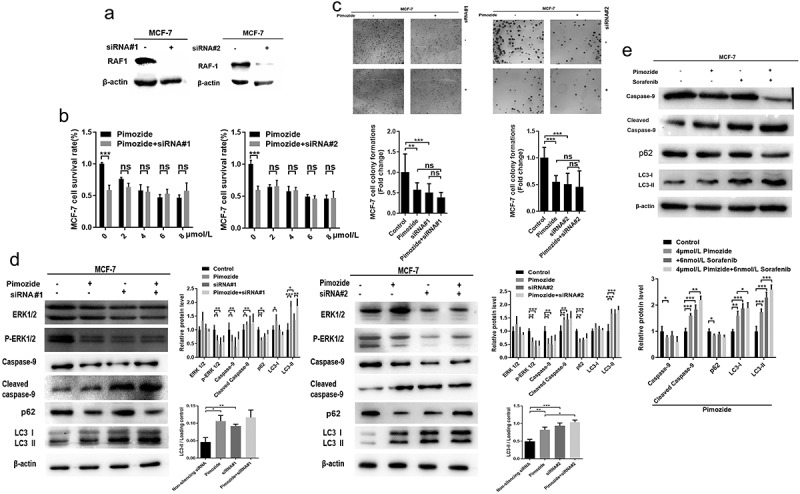
**The effects of RAF1 interference and pimozide treatment on breast cancer cells. (a)** the expression of RAF1 in MCF-7 cells was detected by Western blot after adding siRNA. **(b)** MTT assay was used to detect the effect of different concentrations of pimozide on the proliferation of breast cancer cells (6 × 10^3^ cells/well) without pretreatment or knockdown of RAF1 expression. **(c)** cloning assay was performed to analyze the effects of pimozide (4 μM), siRNA, pimozide, and siRNA on the proliferation of MCF-7 cells (1 × 10^3^ cells), respectively. **(d)** after adding pimozide (4 μM), siRNA, pimozide and siRNA, respectively, the expression of proteins was detected by western blot. **(e)** after adding pimozide (4 μM), Sorafenib (6 nM), pimozide and Sorafenib, respectively, the western blot was used to analyze the expression of proteins. All experiments were performed with DMSO as control and three times in parallel, ns means no statistical significance (*, *p* < .05, **, *p* < .01, ***, *p* < .001).

To examine the role of RAF1 in the anticancer effects of pimozide, we used siRNA to interfere with the effects of RAF1 expression on pimozide-induced apoptosis and autophagy (Figure 5A and D). As shown in Figure 5B and C, after the RAF1 expression was knocked down, the proliferation of MCF-7 cells was significantly inhibited, similar to the effect of pimozide (4 μM). The MCF-7 cells that knocked down RAF1 were treated with pimozide, and its inhibitory effect was similar to knocking down RAF1 alone or adding pimozide.

**Figure 6.**
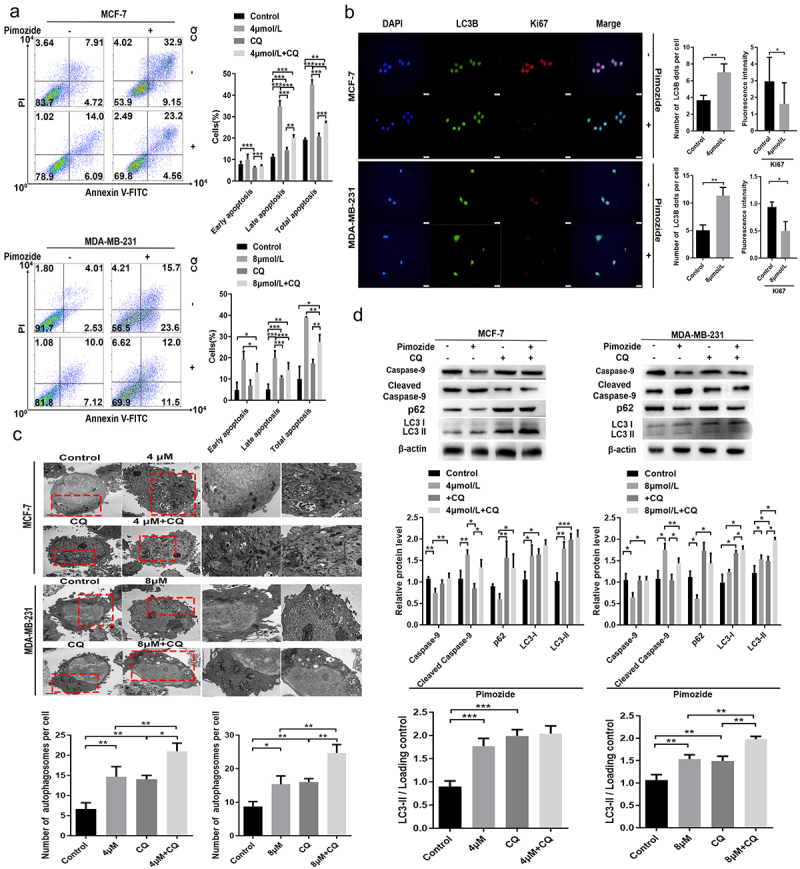
CQ blocks autophagy and repairs pimozide-induced apoptosis of breast cancer cells. (A) flow cytometry was used to examine the effect of pimozide (4 μM or 8 μM) or CQ (50 μM) alone and their combination on apoptosis of breast cancer cells, and cells supplemented with DMSO were used as controls. (B) cell proliferation and apoptosis marker protein expression were detected by immunofluorescence, and cells not treated with pimozide were used as controls. (C) TEM investigates the effects of pimozide (4 μM or 8 μM) or CQ (50 μM) alone and their combination on apoptosis of breast cancer cells, and the red arrow identifies autophagosomes. (D) after autophagy was blocked by CQ (50 μM), the expression of apoptosis and autophagy marker proteins were detected by western blot. All experiments were performed with DMSO as control and three times in parallel, ns means no statistical significance (*, *p*<0.05, **, *p* < .01, ***, *p*<0.001).

Therefore, we further tested its potential mechanism. As shown in Figures 5D and E, 6 nM sorafenib, a RAF1 inhibitor, treated breast cancer cells MCF-7 with similar efficacy to pimozide. Pimozide, sorafenib, and knockdown of RAF1 showed similar results, decreased the phosphorylation level of ERK 1/2, the expression of total ERK 1/2 was unaffected, the expression of full-length caspase-9 was decreased, and the expression of cleaved caspase-9 was increased. However, there was no significant difference in the effect of pimozide treatment on MCF-7 cells that RAF1 was knocked down. In addition, cells were treated with pimozide, sorafenib, or knocked down RAF1 reduced autophagy selective substrate p62 expression and increased autophagy marker protein LC3 I to LC3 II. The addition of pimozide and siRNA was similar to the effect alone. Therefore, these data suggested that the RAF/ERK pathway might play an important role in the anti-cancer effect of pimozide.

## Pimozide promotes cell death with ongoing autophagy

To examine whether the induction of autophagy contributes to pimozide-induced apoptosis, we treated cells with CQ (50 μM) and pimozide for 24 h. We found that while pimozide promoted autophagy in MCF-7 and MDA-MB-231 cells, CQ treatment reversed pimozide-induced autophagy (Figure 6A). We further examined the expression of the autophagy key protein LC3B and the proliferation-associated protein Ki67 by immunofluorescence staining. Consistent with the flow cytometry results, LC3B expression increased while Ki67 expression was down-regulated by pimozide (Figure 6B). We next examined the effects of the administration of pimozide, CQ, or the combination on apoptosis in breast cancer cells. As shown in Figure 6C, pimozide alone induced apoptosis in breast cancer cells, while CQ treatment did not have substantial effects on apoptosis. Upon pimozide and CQ co-treatment of cells, apoptosis was increased compared to levels in cells treated with CQ. However, in co-treated cells in which autophagy was blocked by CQ, the amount of apoptosis induced by pimozide was reduced compared with cells treated with pimozide alone. These results revealed that pimozide promoted autophagy to induce apoptosis in breast cancer cells.

To further determine the mechanism by which pimozide promotes cell death with ongoing autophagy, we examined the expression of the apoptotic initiation protein caspase-9, cleaved caspase-9, and the autophagy key proteins LC3B and p62 by western blot. As shown in Figure 6 6D, after suppressing the autophagic flow, pimozide-induced apoptosis in breast cancer cells was rescued. CQ reduced pimozide-induced caspase 9 cleavage. CQ caused p62 protein accumulation in breast cancer cells. p62 was inhibited in pimozide-treated breast cancer cells. CQ impaired pimozide activation to inhibit p62. Pimozide or CQ alone caused LC3B accumulation, whereas pimozide and CQ co-treatment decreased the conversion of LC3-I to LC3-II. These data indicated that pimozide-induced apoptosis could be reversed by autophagy inhibitors.

## Discussion

Some evidence has shown that antipsychotic drugs are 18 times more likely to produce anticancer activity than random molecules and reports confirmed that the antipsychotic drug pimozide can inhibit the proliferation of cancer cells, including breast cancer cells.^33,34,40–42^ However, the mechanism by which pimozide inhibits breast cancer has been unknown.

In this study, we found that RAF1 might be associated with a poor prognosis in breast cancer. We also constructed a model combining pimozide and RAF1 using Schrodinger software that suggested that pimozide might bind to RAF1. We found that pimozide inhibited the proliferation and colony formation of breast cancer cells. The results of proliferation marker signal Ki67 and colony formation showed that pimozide inhibited the proliferation of breast cancer cells. Flow cytometry, AO-EB staining and immunoblotting showed that pimozide induced apoptosis in breast cancer cells. These results revealed that pimozide influenced both proliferation and apoptosis of breast cancer cells. We examined whether pimozide inhibits breast cancer by regulating RAF/ERK pathway and found that pimozide treatment caused downregulation of RAF1 phosphorylation in breast cancer cells. Previous reports showed that pimozide, as a STAT3 and STAT5 inhibitor, inhibited phosphorylation of ERK 1/2, thereby inhibiting cancer cell proliferation.^43,44^ When RAF1 was silenced, ERK 1/2 phosphorylation was subsequently inhibited, and apoptosis and autophagy were promoted in breast cancer cells, indicated that RAF1 signaling functions to regulate apoptosis and autophagy in breast cancer cells. Our results showed that pimozide inhibited RAF1 phosphorylation. Therefore, our work suggests that pimozide functions by modulating RAF1 signaling. Combined with previous findings, it indicated that there might be multiple pathways for pimozide to modulate the same biological signaling. ERK 1/2 is a downstream protein of RAF1, and regulation of RAF1 expression may affect the activation of ERK 1/2 signaling. We found that pimozide treatment resulted in reduced RAF1 phosphorylation and thus inhibition of phosphorylation of RAF1 may play an important role in the anticancer activity of pimozide. Our results suggest that pimozide might exert its anticancer activity in inhibiting the proliferation of breast cancer cells by blocking the RAF/ERK signaling pathway. Bertolesi et al. demonstrated that pimozide inhibited MCF-7 cell growth by interfering with T-type Ca^2+^ channels.^13^ which are associated with changes in the mitochondrial membrane potential.^45^ Our JC-1 assay results demonstrated that pimozide treatment caused an increase in the number of cells with reduced mitochondrial membrane potential. We speculate that these results may be attributed to pimozide inhibiting Ca^2+^ channels and inducing changes in the expressions of mitochondrial membrane permeability-related proteins Bcl-2, Bcl-xl, and Bax.

Previous studies demonstrated that activation of the RAF1 signal promotes autophagy.^46^ Interestingly, ERK 1/2 is upregulated in response to mTOR signaling, while mTOR was shown to inhibit autophagy.^47^ Therefore, we investigated the effect of pimozide on autophagy in breast cancer cells and found that pimozide promoted autophagy in breast cancer cells. Notably, while pimozide did not impact RAF1 and ERK 1/2 protein expression, pimozide down-regulated both RAF1 and ERK 1/2 phosphorylation levels, suggesting that other factors may be involved in the regulation of autophagy. Furthermore, mTOR expression was not affected by pimozide, likely as other signals are involved in the regulation of mTOR, such as the p53 pathway.^48^ Since previous studies showed that RAF1 can form a complex with phosphodiesterase, which hydrolyzes cAMP, while cAMP can down-regulate RAF1 expression depending on the PKA pathway and cAMP can promote autophagy,^36,37,49^ we tested the expression of cAMP by FRET and found that pimozide treatment increased the expression of cAMP in breast cancer cells, which may lead to enhanced autophagy in breast cancer cells. Interestingly, overexpression of cAMP regulated the activity of the PKA pathway, thereby promoting apoptosis of cancer cells.^50^ We synthesized siRNA to interfere with the expression of RAF1. We found that RAF1 was knocked down or treated with pimozide to promote apoptosis and autophagy in breast cancer cells, and there was no significant difference between the two treatments. Interestingly, knockdown of RAF1 resulted in reduced expression of p-ERK 1/2, and caspase-9 was rescued by pimozide. This may be due to the up-regulation of cAMP in cells by pimozide, which has been shown to upregulate phosphorylation of ERK, leading to the combination of pimozide and siRNA to counteract the down-regulation of ERK 1/2 phosphorylation induced by siRNA alone.^39^ Caspase-9 also produces a cascade reaction as a downstream signal. Since autophagy is associated with apoptosis, we examined the effect of CQ-blocked autophagy on pimozide-induced apoptosis. The data showed that CQ reversed pimozide-induced apoptosis, similar to previous results in glioblastoma cells.^37^ It is worth noting that pimozide could down-regulate ULK1-related autophagy by inhibiting USP1.^51^ We speculated that pimozide may be more intense autophagy mediated by cAMP. Pimozide promoted the degradation of ID1 mediated by USP1, which led to interference in cancer progression.^52^ Reports had shown that ID1 could activate the RAF/MEK signaling pathway in nasopharyngeal carcinoma cells and glioblastoma,^53,54^ which might reveal that ID1 had a similar effect in breast cancer cells. The addition of the neuroleptic drug pimozide to inhibit ID1 expression enhanced the cytotoxic effects of drug therapy on glioma cells and non-small cell lung cancer,^53,55^ but the data showed that pimozide had an antagonistic effect on the efficacy of other drugs in non-small cell lung cancer.^56^ It has been the previous study presented that pimozide inhibited the AKT signaling pathway and epithelial-mesenchymal transition and cell migration in MDA-MB-231 breast cancer cells, and downregulated the expression of MMPs.^57^ However, in a difference from previous studies, our data demonstrated that pimozide inhibited the phosphorylation of RAF1 while exerting an effect on apoptosis and autophagy in breast cancer cells and that the transient fluctuating rise in cAMP might be associated with cellular autophagy. Hence, our work reveals a potential pathway for pimozide to induce apoptosis in breast cancer. In the follow-up work, we will study the regulation of unknown regulatory factors by pimozide in breast cancer cells, resulting in the blockage of cross-linking between RAF1 and the Hippo pathway. In addition, the elevated level of cAMP expression, whether it means that the PKA pathway is also activated accordingly will also be our focus.

**Figure 7.**
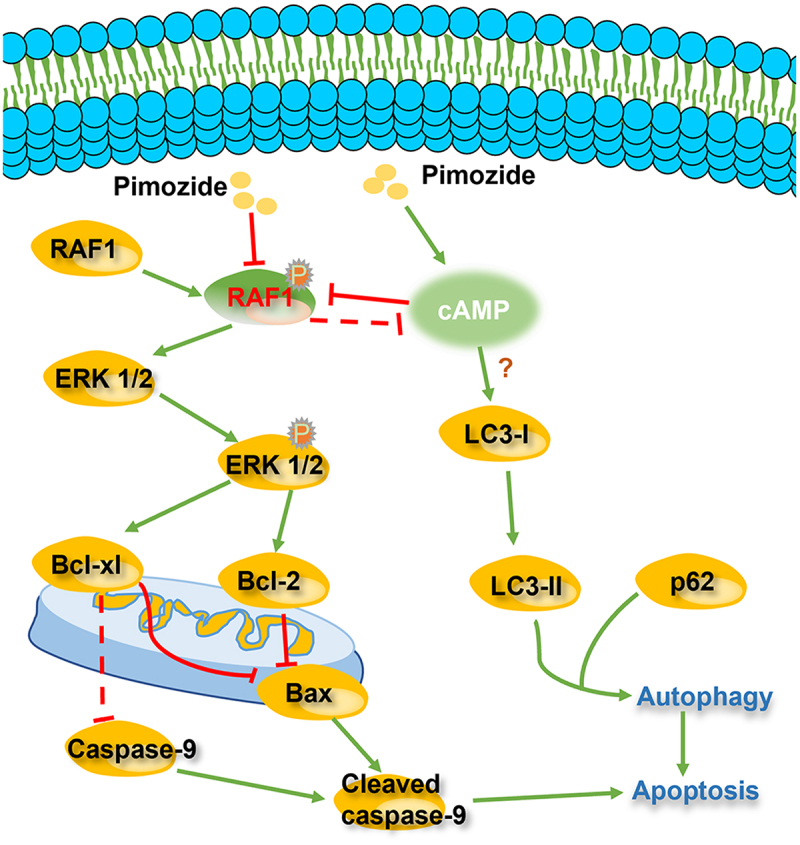
Pimozide exerts a possible mechanism of action to inhibit breast cancer activity..

In summary, our results revealed that pimozide inhibits cell proliferation by inducing apoptosis and autophagy in breast cancer cells (Figure 7). We found that pimozide inhibits the colony formation activities of breast cancer cells. Moreover, we demonstrated that pimozide inhibits breast cancer by down-regulating the RAF/ERK pathway. The Hippo pathway was not regulated by pimozide in breast cancer cells. Pimozide might promote autophagy in breast cancer cells by up-regulating cAMP, rather than by inhibiting mTOR signaling. The autophagy inhibitor CQ rescued apoptosis induced by pimozide. These data suggested that pimozide may induce apoptosis of breast cancer cells by promoting autophagy. Our study provided evidence that pimozide could be a potential drug for the clinical therapy of breast cancer.

## Materials and methods

### Reagents

Pimozide was purchased from J&K Scientific (P447800). Chloroquine (CQ) was purchased from Solarbio (IC4440). Sorafenib was purchased from Beyotime Biotechnology (SC0236). The caspase-6 activity assay kit and diamidino-phenyl-indole were purchased from Solarbio (BC3860). Epac-SH187 was purchased from Vector Biolabs. MTT was purchased from Solarbio (M8180). Antibodies against RAF1, phospho-ERK 1/2 (Thr202/Tyr204), and Bax were purchased from Elabscience (E-AB-60020, E-AB-70310, E-AB-10049). Antibodies against ERK 1/2, phosphor-RAF1 (Ser338), cleaved caspase-9, Bcl-xl, and Hippo signaling antibody sampler kit were purchased from Cell Signaling Technology (9102, 9427, D8I9E, 54H6, 8579). Antibodies against mTOR, phospho-mTOR (phospho S2448), LC3B, and SQSTM1/p62 were purchased from Abcam (ab32028, ab109268, ab51520, ab109012). Antibodies against Bcl-2 and β-actin were purchased from Bioss (bs-0032 R, bs-0061 R/bsm -33,036 M). Antibodies against Ki-67 were purchased from Zhongshanjinqiao Biological Technology Ltd (TA500265).

## Tissue samples

The samples were purchased from Shanghai Outdo Biotech Co., LTD. The samples were routinely processed with 10% buffered formalin fixation and paraffin-embedded. The appropriate paraffin block was selected for this study.

## Immunohistochemistry (IHC)

IHC used a 2-step plus poly-HRP anti-rabbit/mouse IgG detection kit (Elabscience, E-IR-R213). After the slides were washed by dewaxing and phosphate-buffered saline, the antigen was repaired by immersing the slides in 0.01 M sodium citrate buffer at 80°C, and then the slides were cooled at room temperature for 1 hour. After dipping in PBS, 3% hydrogen peroxide covers the slides for 15 minutes at room temperature in a humid chamber. The slides were incubated with RAF1 antibody in a humid chamber overnight at 4°C. After washing with PBS, the slides were incubated with the secondary antibody for 1 hour, stained with 3, 30-diaminobenzidine tetrahydrochloride (Zhongshanjinqiao, ZLI-9018), and then Mayer’s hematoxylin counterstained. The rabbit IgG isotype was used as a control to show negative results. The staining of the entire tissue sample was evaluated at high magnification (×20, BX53, Olympus Corporation, Tokyo, Japan).

## Cell culture and treatment

The human breast cell lines MCF-7 and MDA-MB-231 were donated by Professor Changliang Shan of Nankai University and were cultured in high glucose Dulbecco’s Modified Eagle’s Medium (HyClone, SH30022.01) supplemented with penicillin (100 unit/ml), streptomycin (100 μg/ml), and 10% fetal bovine serum (Gemini, 900–108) in an atmosphere of 5% CO_2_ at 37°C. Cells were exposed 24 h to each concentration of pimozide and CQ.

## Drug virtual docking analysis

Download the RAF1 protein crystal structure (3OMV) from the Protein Data Bank (http://www.rcsb.org/), this crystal structure was unmodified or not combined with other molecules, derived from the complete crystal structure of homo sapiens. The pimozide structural formula (CAS number 2062-78-4) was downloaded at Drugbank (https://www.drugbank.ca/). Dock pimozide and RAF1 using Schrodinger 2017 (Schrodinger, New York, USA) according to the guidelines provided by the software manufacturer.

## Cell viability assay

The cytotoxic potential of pimozide in MCF-7 and MDA-MB-231 cells was determined by MTT assay. Cells were seeded into 96-well plates at a cell density of 6 × 10^3^ cells/well in the growth medium. Cell growth was then assayed by the addition of 20 μL of MTT to each well, and the plate was incubated at 37°C for 4 h. The reaction was stopped by the addition of 200 μL DMSO (Applichem, A3672.0250). Optical density was measured at 570 nm.

For cell counting, 5 × 10^4^ cells were seeded in 6-well plates, and MCF-7 and MDA-MB-231 cells were treated with 4 μM and 8 μM pimozide, respectively. Surviving cells were counted under the microscope (×10, BX53, Olympus Corporation, Tokyo, Japan) after 1, 2, 3, and 4 days of drug treatment.

## Colony formation assay

Cells were seeded in 6-well plates and treated with DMSO or pimozide in triplicate for 14 days. Colonies were fixed with the cold MeOH, stained with 0.2% crystal violet, and counted manually.

## Immunofluorescence

The 1 × 10^3^ cells were seeded in 6-well plates and treated with DMSO or pimozide for 12 h, and then prefixed in MeOH for 30 min at room temperature, followed by permeating in 0.5% Triton X-100 for 20 min. The sections were then rinsed thrice in PBS and blocked with 5% skim milk at room temperature for 1 h. After washing thrice with PBS, the slides were incubated at 4°C overnight with primary antibodies. The sides were then rinsed thrice in PBS, incubated for 2 h at room temperature with fluorescence-labeled secondary antibody, and treated with DAPI at room temperature for 10 min. Finally, the sections were observed with a fluorescence microscope (Olympus, Tokyo, Japan) after washing with PBS thrice.

## Acridine orange and ethidium bromide (AO-EB) staining

The 1 × 10^3^ cells were seeded in a 6-well plate and made into a slide. Washed twice with PBS to remove residual medium and unattached cells, and added new PBS. Add 20 ul of AO-EB (Solarbio, CA1140) working solution per ml of PBS. After standing at room temperature for 5 min, it was washed twice with PBS to remove the residual fluorescent dye. Add a new PBS to observe under a fluorescence microscope.

## Caspase activity assay

The cells were collected in a centrifuge tube, and the supernatant was discarded after centrifugation. The 100 μL of reagent 2 was added to the centrifuge tube, and the pellet was resuspended. Then let it stand on ice for 15 min. Centrifuged it at 15,000 g for 15 min at 4°C. Then performed operations according to the manufacturer’s instructions, and detected the activity of caspase 6 at 405 nm.

## Flow cytometry analysis for cell apoptosis

Annexin V-FITC Apoptosis Detection Kit (BD 556,547) was used to evaluate the percentage of apoptotic cells. MCF-7 and MDA-MB-231 cells were treated with pimozide for 24 h. Then, 5 × 10^5^ treated cells were centrifuged and re-suspended in cold 1 × binding buffer, after which 1.25 μl of Annexin V-FITC and 10 μl of propidium iodide were added and the suspension was analyzed by flow cytometry (Beckman Coulter, CA, USA).

## JC-1 analysis

The mitochondrial membrane potential was detected by using JC-1 staining. Cells were harvested, washed with PBS, and incubated with 5 µM JC-1 (Beyotime, C2006) for 30 minutes in the incubator. Then, the cells were washed and data were analyzed using Flowjo software.

## RNA interference

Predesigned pools of siRNA oligonucleotides (Guangzhou RiboBio Co., Ltd.) were used for silencing the expression of RAF1 (target sequence: GCACCAAAGTACCTACTAT^#1^, GCAGCAGCCTCTACAAACA^#2^). The MCF-7 cells were transfected with 50 nM siRNA against RAF1. The cells were incubated for 48 h in the growth medium containing the transfection complexes. Transfections with siRNA were performed in serum-free DMEM following the manufacturer’s instructions.

## FRET analysis

The cells were seeded in a 6-well plate, and the cAMP fluorescent probe Epac-SH187 was transfected with adenovirus, and a 2 μl virus was added to each well. After 12 hours, the OptoLED fluorescence imaging system (Cairn Research Ltd, UK) and the equipped CoolSNAP HQ2 digital CCD camera (Photometrics, Tucson, AZ, USA) and a beam splitter (DV2, Photometrics, USA) connected to the Nikon inverted microscope were used to detect cAMP changes in living cells in real-time.

## Monodansylcadaverine (MDC) staining

Cells were seeded on sterile coverslips in 6-well plates and incubated in pimozide or DMSO for 24 hours. After the incubation, the cells were digested with trypsin, centrifuged at 800 g for 5 min, and the cells were collected. The cells were washed once with 300 μl of 1×wash buffer, and the supernatant was discarded. The cells were resuspended by adding 500 μl of 1×wash buffer, and the cell concentration was counted and adjusted to 10^6^/ml. An appropriate amount of 90 μl of the cell suspension was added to a new tube, and 10 μl of MDC Stain (Solarbio, G0170) was added. Stain at room temperature for 30 min. After centrifugation at 800 g for 5 min, the cells were collected, the cells were washed twice with 300 μl of 1×wash buffer, and the supernatant was discarded. The cells were resuspended by adding 100 μl of collection buffer and added dropwise to the glass. Observe and photograph under a fluorescence microscope (×20, BX53, Olympus Corporation, Tokyo, Japan).

## Transmission electron microscopy (TEM) analysis

The 1 × 10^7^ Cells were treated with pimozide and CQ for 24 h. After treatment, the cells were harvested and fixed with 2.5% glutaraldehyde overnight at 4°C. After washing with 0.1% sodium cacodylate buffer, the cells were post-fixed with 1% osmium tetroxide for 30 min, stained with 2% uranyl acetate at 4°C, dehydrated in a graded ethanol series, and embedded in spur resin. Ultra-thin (70 nm) sections were cut on a Reichert Ultra cut microtome, post-stained with uranyl acetate and lead citrate, and washed with water. The sections were examined by a transmission electron microscope JEM-2100 (JEOL, Musashino, Akishima, Tokyo) operated at 200 kV.

## Western blot

The total proteins were collected from MCF-7 and MDA-MB-231 cells cultured with various concentrations of pimozide for 24 hours. Protein concentrations were analyzed by the BCA Protein Assay Kit (Solarbio, PC0020). Then, the proteins were separated by 10% SDS-PAGE and transferred onto PVDF membranes (EMD Millipore, Billerica, MA, USA). The membranes were blocked with 5% nonfat milk for 1 hour at room temperature followed by incubation overnight at 4°C with a primary antibody (RAF1, p-RAF1, ERK 1/2, p-ERK 1/2, Bcl-2, Bcl-xl, Bax, mTOR, p-mTOR, β-actin) at a dilution of 1:1,000 or LC3B, p62 at a dilution of 1:3,000. Subsequently, the membranes were washed three times with PBST and incubated with the appropriate HRP-conjugated secondary antibody (1:2500) for 1 hour at room temperature. Protein bands were visualized in the darkroom and quantitated using the ImageJ software. β-actin was used as an internal control.

## Statistical analysis

All statistical analyses were performed using GraphPad Prism 5.0 (San Diego, CA). Values were presented as the ratio ± SD of the control. The paired student’s t-test was used to analyze statistical significance. *p* < .05 was considered to indicate statistical significance. Each experiment was performed in triplicate.

## Supplementary Material

Supplemental material.docx

## Data Availability

The data that support the findings of this study are available from the corresponding author, [S.L], upon reasonable request.
