# Supplementary material for: The antipsychotic drug pimozide promotes apoptosis through the RAF/ERK pathway and enhances autophagy in breast cancer cells
Source: Cancer Biol Ther. 2024 Feb 14;25(1):2302413. doi: 10.1080/15384047.2024.2302413 (PMC10878017; doi:10.1080/15384047.2024.2302413)
Supplement: Supplemental material.docx [file KCBT_A_2302413_SM0257.docx]

**Supplementary figure legends**

**Figure S1. The Kaplan-Meier database was used to analyze the relationship between the protein expression or mRNA level of RAF1 and overall survival and distant metastasis-free survival in breast cancer patients with PR (+/-) or ER (-) breast cancer.**

**Figure S2. The effect of pimozide on the expression of Hippo pathway proteins YAP/TAZ, MST1, MST2 in breast cancer cells.**

**Figure S1.**

**
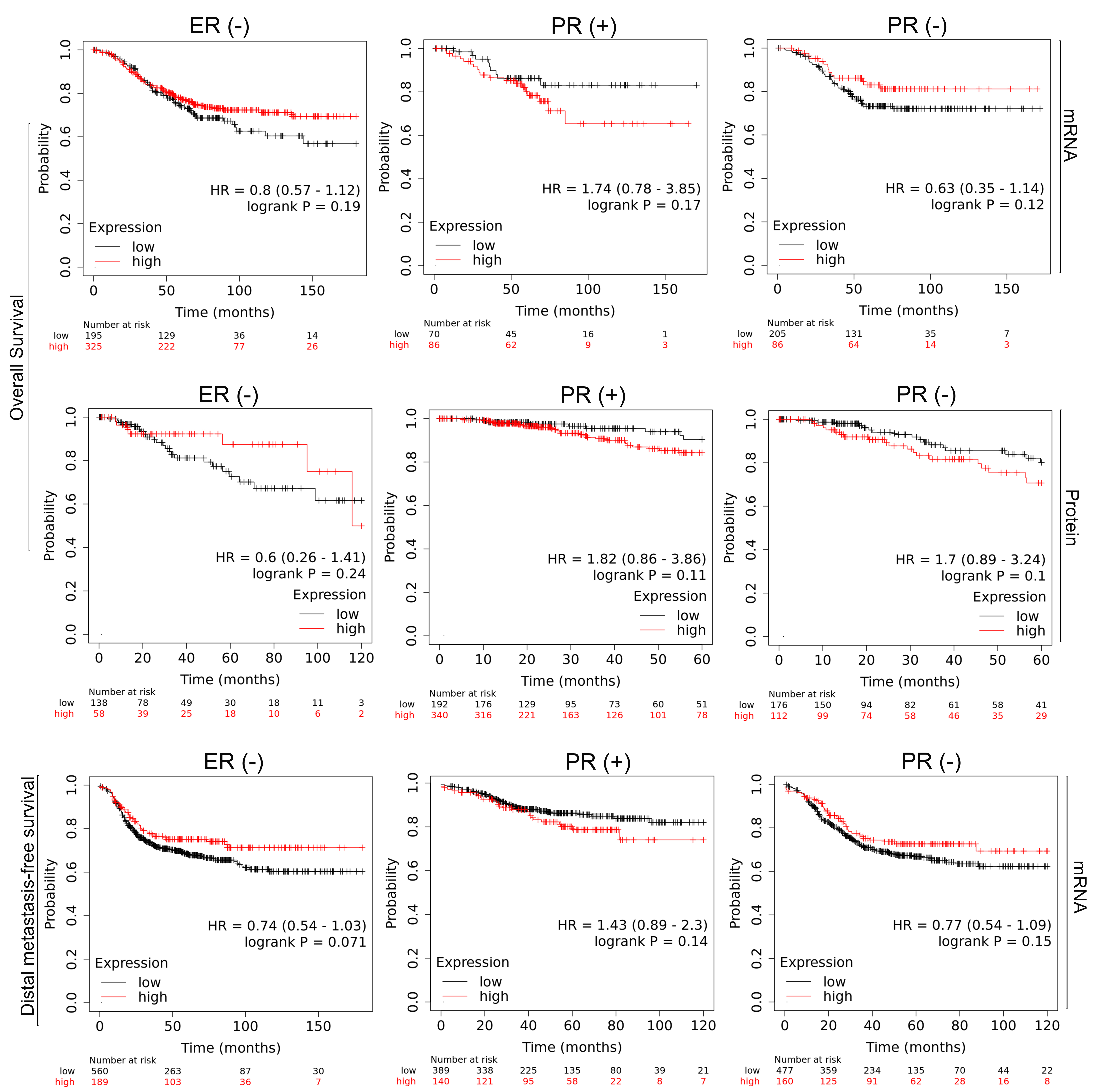
**

**Figure S2.**

**
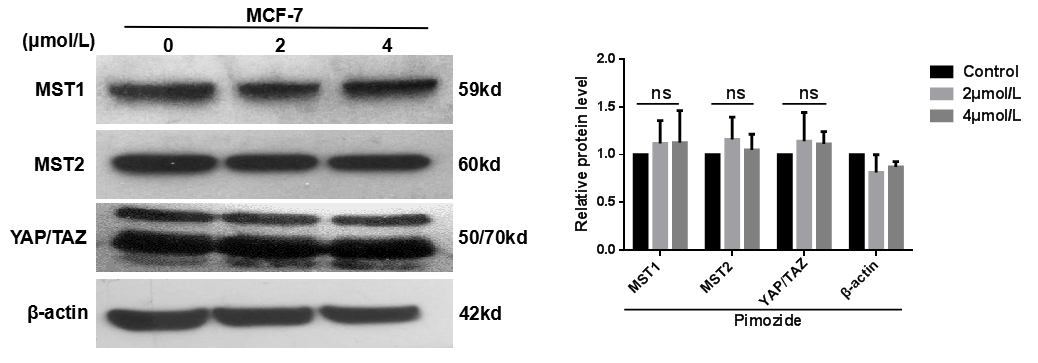
**
